# Is three-dimensional ultrasonography a valuable diagnostic tool for patients with ovarian cancer? Systematic review and meta-analysis

**DOI:** 10.3389/fonc.2024.1404426

**Published:** 2024-07-08

**Authors:** Yuan Liu, Qingdong Zhang, Fang Zhang, Meiyan Liu, Jun Zhang, Xiaoli Cao, Weihong Yin

**Affiliations:** ^1^ School of Medical Imaging, Binzhou Medical University, Yantai, Shandong, China; ^2^ Department of Ultrasonography, Affiliated Yantai Yuhuangding Hospital of Qingdao University, Yantai, Shandong, China; ^3^ Department of Ultrasonography, Yantai Affiliated Hospital of Binzhou Medical University, Yantai, Shandong, China; ^4^ Department of Scientific Research and Teaching, Yantai Affiliated Hospital of Binzhou Medical University, Yantai, Shandong, China

**Keywords:** ovarian cancer, three-dimensional ultrasonography, three-dimensional power Doppler ultrasonography, meta-analysis, diagnose

## Abstract

**Introduction:**

This paper was to assess the diagnostic performance and clinical value of three-dimensional ultrasonography (3DUS), three-dimensional ultrasonography power Doppler (3DPD), and 3DUS combined with 3DPD in ovarian cancer (OC).

**Methods:**

The study was registered with PROSPERO (CRD 42023405765). PubMed and Web of Science were searched from inception to 25 January 2022, and reference lists of potentially eligible studies were also manually searched. Patient and study characteristics were extracted by two independent reviewers. Any discrepancies were addressed through discussion. The sensitivity, specificity, positive and negative likelihood ratio (PLR and NLR, respectively), and the area under the receiver operating characteristic curve (AUC) were pooled separately.

**Results:**

We retrieved 2,566 studies, of which 18 were finally enrolled, with 2,548 cases. The pooled sensitivity, specificity, PLR, NLR, and AUC for 3DUS were 0.89 (95% CI: 0.85–0.93), 0.93 (95% CI: 0.88–0.96), 13.1 (95% CI: 7.3–23.4), 0.11 (95% CI: 0.08–0.16), and 0.90 (95% CI: 0.87–0.93), respectively. The pooled sensitivity, specificity, PLR, NLR, and AUC for 3DPD were 0.90 (95% CI: 0.80–0.95), 0.85 (95% CI: 0.71–0.92), 5.8 (95% CI: 3.0–11.2), 0.12 (95% CI: 0.06–0.24), and 0.94 (95% CI: 0.91–0.96), respectively. The pooled sensitivity, specificity, PLR, NLR, and AUC for 3DUS combined with 3DPD were 0.99 (95% CI: 0.73–1.00), 0.95 (95% CI: 0.85–0.99), 21.9 (95% CI: 6.1–78.9), 0.01 (95% CI: 0.00–0.37), and 0.99 (95% CI: 0.98–1.00), respectively.

**Conclusions:**

3DUS, 3DPD, and 3DUS combined with 3DPD are promising diagnostic tools for OC, alongside elevated sensitivity and specificity. However, the combination of 3DUS and 3DPD techniques has higher diagnostic efficiency.

**Systematic Review Registration:**

https://www.crd.york.ac.uk/PROSPERO/, identifier CRD 42023405765.

## 1 Introduction

Ovarian cancer (OC) is a major concern for women’s health, accounting for 3.4% of female cancer cases and with a mortality rate of 4.7% (1). Patients at the International Federation of Gynecology and Obstetrics (FIGO) stage I have a high 5-year survival rate of 90%, but those at FIGO stage IV have a high 5-year survival rate of no more than 10% (2). Nonetheless, a significant proportion, approximately 70% of OC patients do not receive a diagnosis until the disease has progressed to advanced stages. This delay in diagnosis is primarily due to its asymptomatic nature in the initial stages and its deep location within the pelvis (3). This underscores the critical importance of ongoing research efforts to develop early and reliable diagnostic techniques for OC.

The gold standard for OC diagnosis is histopathology (4), which, however, is invasive. As alternatives, non-invasive techniques such as biochemical and imaging examination, including carbohydrate antigen 125 (CA125), human epididymis protein 4 (HE4), two-dimensional ultrasonography, computer tomography (CT), and magnetic resonance imaging (MRI) are commonly used in preoperative assessment of OC. However, each of these options presents disadvantages. For instance, elevated CA125 levels may also be detected in other malignancies and certain benign gynecological conditions (5). HE4 levels are susceptible to factors such as body mass index and smoking (6). CT scans expose patients to high doses of ionizing radiation, particularly for repeated scans, while MRI scans are expensive and not widely accessible (7). Ultrasonography has become one of the preferred diagnostic methods for distinguishing OC due to its non-invasive, simple, repeatable, cost-effective, and real-time features. The International Ovarian Tumor Analysis has introduced simple ultrasound-based rules, logistic regression model, and assessment of different neoplasms in the adnexa (ADNEX model) for preoperative evaluation of OC patients (8). Nevertheless, two-dimensional ultrasonography is heavily dependent on the operator and presents limited sensitivity (9).

Three-dimensional ultrasonography (3DUS) offers solutions to several major limitations of conventional two-dimensional ultrasound (10). It can obtain and store the volume of a region of interest (ROI). It requires a few volumetric scans to obtain a comprehensive image of the entire tissue. These images can be easily reviewed and re-examined electronically from various angles and planes, allowing different expert operators to assess the tissue independently of the initial sonographer. This significantly reduces the reliance on the original sonographer. Tissue vascularization in the ROI can also be assessed using three-dimensional ultrasonography power Doppler (3DPD), which helps generate three-dimensional reconstructions of the vascular network and is particularly significant in the field of oncology. The vascular structure in malignant tumors is different from that of benign ones, with tumor tissue typically displaying a higher microvessel density, a phenomenon referred to as angiogenesis (11). Furthermore, power Doppler has been shown to be 3 times more sensitive than color Doppler in detecting low-flow velocities (12), making it an important tool for assessing blood flow in tissues.

Studies have examined the accuracy of both 3DUS and 3DPD in detecting OC, however, yielding conflicting results (13–16). We conducted a meta-analysis and systematic review to assess the efficacy of 3DUS, 3DPD, and 3DUS combined with 3DPD in distinguishing between benign and malignant ovarian tissue.

## 2 Materials and methods

### 2.1 Search strategy and selection criteria

Our study was registered with PROSPERO (CRD 42023405765). PubMed and Web of Science were systematically searched for studies on the diagnostic value of 3DUS and 3DPD in OC between inception and 25 January 2022. The search strategy is shown in **Supplementary Material S2**. The reference lists of potentially eligible studies and prior systematic reviews were manually examined.

The inclusion criteria encompassed the following: (1) individuals diagnosed with OC with no limitation on duration of illness, other diseases, or complications; (2) studies examining the diagnostic value of 3DUS, 3DPD, and 3DUS combined with 3DPD; (3) postoperative histopathology as the diagnostic standard; (4) studies providing sufficient data to construct the 2 × 2 contingency table, including false positives, true positives, false negatives, and true negatives; (5) no limitation on study type.

Studies were excluded for the following reasons: (1) utilization of index tests in conjunction with other diagnostic methods; (2) emphasis on 3DPD vascular indexes; (3) not taking postoperative histopathology as the gold standard; (4) insufficient data provided in the studies to obtain 2 × 2 contingency table directly or indirectly; (5) abstracts, reviews, case reports, animal experiments, correspondences, conferences, and lectures.

### 2.2 Data collection and quality assessment

Data collection and quality assessment were independently conducted by two researchers (L.Y., Z.Q.D.). Any discrepancies were addressed through group discussion after reviewing the full text of the available articles.

The name of the first author, publication year, country, publication type, type of study, ultrasound machine, masses type, and sample size (ultrasound and gold standard) were extracted from each included study. The count of true positives, false positives, true negatives, and false negatives was independently documented by each investigator.

The methodological quality of the included studies was assessed using QUADAS (quality assessment of studies of diagnostic accuracy) (17). The evaluation criteria for each item on the checklist were customized to suit our review (18). If additional information was required, we would contact the corresponding authors.

### 2.3 Statistical analysis

The true positives, false negatives, false positives, and true negatives among OC patients were calculated according to study classification to compute sensitivity and specificity and the corresponding 95% confidence interval (CI).

An exact binomial rendition (19) of the bivariate mixed-effects regression model developed by van Houwelingen (20, 21) was employed for comprehensive data analysis, which was suitable for meta-analysis of treatment trials and was modified for comprehensive analysis of diagnostic test data (22, 23). This model preserves the original two-dimensional nature of the data without transforming the sensitivity and specificity of individual studies into a single measure of diagnostic accuracy. It considers any correlation between sensitivity and specificity. Based on this model, the mean logit sensitivity and specificity with their standard errors and 95% CIs were estimated. Inter-study variability in logit sensitivity and specificity and their covariance was also calculated. These data were inversely concerted to the original receiver operating characteristic (ROC) curve to obtain summary sensitivity, specificity, and diagnostic odds ratios. Furthermore, a hierarchical summary ROC curve was plotted for 3DUS and 3DPD, with summary points for sensitivity and specificity and a 95% confidence ellipsoid on the curves (two-dimensional CI).

I² statistics was utilized to assess heterogeneity (24). The Cochrane Q test was utilized to obtain I², where I² <50% indicated moderate heterogeneity, and thus a fixed-effects model was used to pool the effect measures. Conversely, I² >50% indicated high heterogeneity, so a random-effects model was employed. In cases of heterogeneity, meta-regression, and sensitivity analysis were performed to determine the potential sources of heterogeneity. Based on the characteristics of each literature, specific covariates and criteria were set as follows: ① sample size (size50): sample size ≥50 was set as 1, while sample size <50 was set as 0; ② included standard of OC (subject): no special descriptions was set as 1, while special descriptions of included standard was set as 0; ③ ultrasound machine manufacturer (machine): GE Voulson machines was set as 1, while the machine from other manufacturers was set as 0.

To assess publication bias, a funnel plot of the log of effective sample size versus the diagnostic odds ratio was generated, and a regression test was conducted to assess asymmetry (25).

Cohen’s κ statistics were calculated by two investigators to assess methodological quality.

The MIDAS module of STATA (version 15.0) was used to summarize the pooled estimates and to perform the meta-regression and graphing.

### 2.4 Compliance with ethics guidelines

This article is based on previous studies and does not involve any human participants or animals in any included studies.

## 3 Results

**Figure 1:**
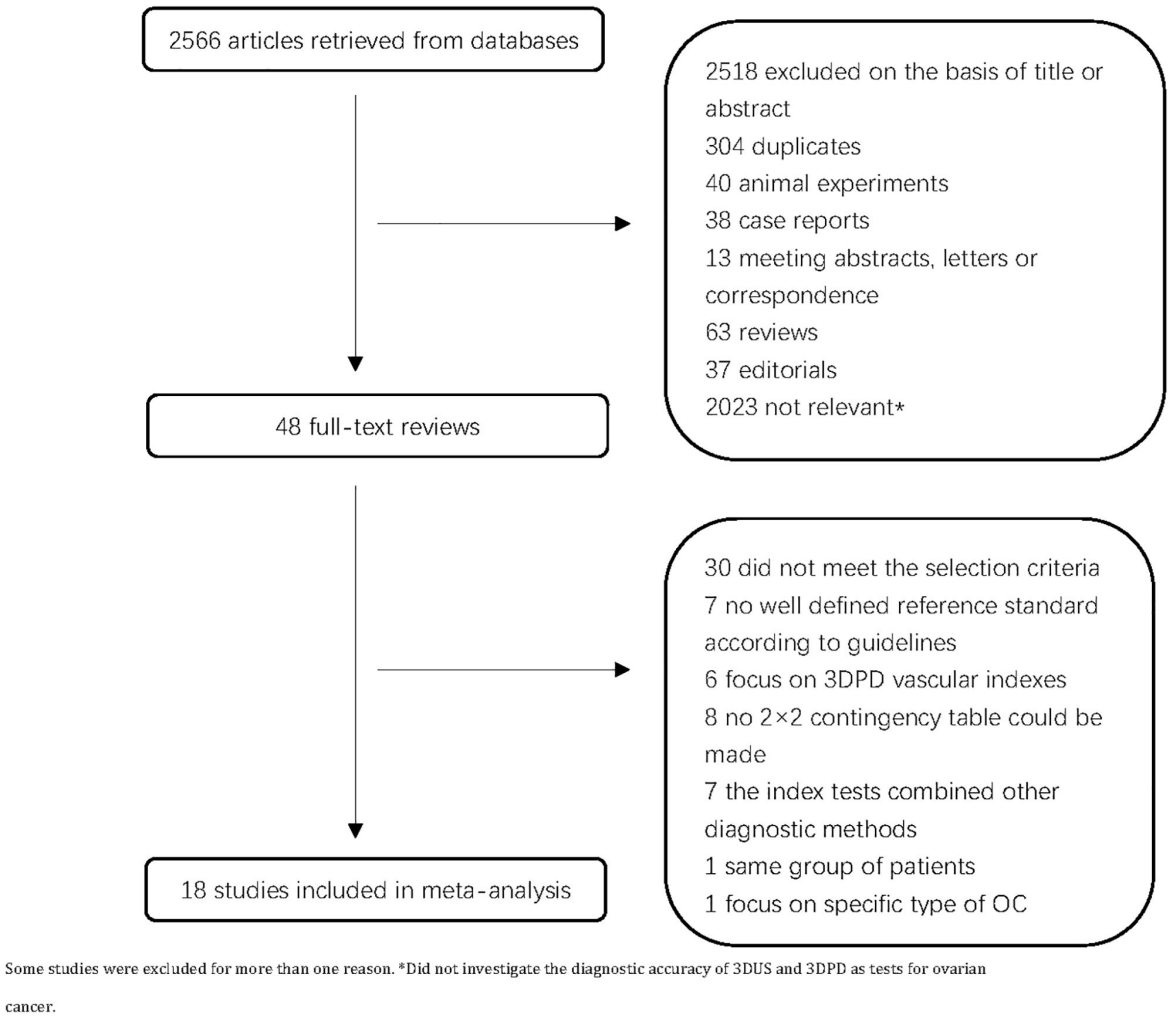
Flow diagram of the study selection process.

A total of 2,566 articles were retrieved. Following a review of the titles and abstracts, we excluded 2,518 articles. After full-text assessment, seven articles were excluded due to the lack of well-defined reference criteria according to guidelines, seven articles were excluded because the index tests were combined with other diagnostic methods, six articles on 3DPD vascular indexes were excluded, eight articles were excluded due to the lack of 2 × 2 contingency table, one study was excluded due to the patient with the same sequence, and another one study was excluded because it focused on specific type of OC. Finally, 18 primary studies were enrolled (**Figure 1**). **Table 1** shows the main characteristics of the 18 included studies (13–16, 26–39)with 1652 ovarian lesions. The sample size varied from 20 to 318. The number of analyzed ovarian lesions was counted, not the number of patients. There were seven studies on 3DUS, eight studies on 3DPD, and six studies on 3DUS combined 3DPD. Histopathologic diagnoses were regarded as the gold standard in all studies (**Table 1**).

**Table 1:** Study characteristics.

Study	Country	Publication type	Declared design	Ultrasound device	Masses type	n1	n2	Modality
Alcazar, J.L., 2008	Spain	journal article	retrospective	GE Voluson 730 Expert	··	39	39	3DPD
Alcazar, J.L., 2005	Spain	journal article	prospective	GE Voluson 730-pro/SonoAce SA-9900	complex masses	69	69	3DPD
Alcazar, J.L., 2003	Spain	journal article	prospective	SonoAce SA-9900	complex masses	44	44	3DUS
Alcazar, J.L., 2007	Spain	journal article	prospective	GE Voluson 730	··	82	82	3DUS
Alcazar, J.L., 2012	Spain	journal article	prospective	GE Voluson 730 Expert	··	99	69	3DUS
Cohen, L.S., 2001	America	journal article	prospective	GE Voluson 530D	complex masses	71	71	3DUS and 3DPD
Dai S.Y., 2008	Japan	journal article	prospective	GE Voluson 730 Expert	complex masses	36	36	3DPD
Geomini, P.M., 2006	the Netherlands	journal article	prospective	GE Voluson 730	··	181	181	3DUS and 3DPD
Hata, T., 1999	Japan	journal article	prospective	Aloka SSD-1700	··	20	20	3DUS
Kalmantis, 2013	Greece	journal article	prospective	GE Voluson 730	··	318	318	3DUS and 3DPD
Kupesic, S., 2000	Croatia	journal article	prospective	GE Voluson 530	complex masses	45	45	3DPD
Kurjak, A., 1999	Croatia	journal article	prospective	GE Voluson 530	··	120	120	3DUS and 3DPD
Kurjak, A., 2001	Croatia	journal article	prospective	GE Voluson 530D	··	251	251	3DUS and 3DPD
Kurjak, A., 2000	Croatia	journal article	prospective	GE Voluson 530	··	90	90	3DUS and 3DPD
Laban, M.,2007	Saudi Arabia	journal article	prospective	GE Voluson 730 Pro	complex masses	50	50	3DUS and 3DPD
Pascual, M.A., 2011	Spain	journal article	retrospective	GE Voluson 730 Expert	··	41	31	3DUS
Perez-Medina, T., 2013	Spain	journal article	prospective	GE Voluson 730	complex masses	72	72	3DUS and 3DPD
Testa, A.C., 2005	Italy	journal article	prospective	Esaote Technos MP	solid masses	24	24	3DPD

n1, ovarian cancer cases; n2, cases with histopathology results; TP, true positive; FP, false positive; TN, true negative; FN, false negative.

3DUS and 3DPD, which were used to distinguish benign from malignant ovarian lesions, examined the structure of the ovarian wall, shadowing, solid parts, septa, presence of the peritoneal fluid, surface, relationship with surrounding structure, vessel architecture, and branching pattern (22). Malignant tumors were classified postoperatively according to the FIGO system (40).

**Supplementary Material S1** shows the methodological quality of the included studies according to the QUADAS checklist. Most studies (83.3%, 15/18) were of high quality (QUADAS score ≥ 8), two studies had a QUADAS score of 7, and one had a QUADAS score of 5.

### 3.1 3DUS

Nine studies on 3DUS were included, with 749 lesions. Most studies were prospective, with only one retrospective study (37). Five articles were limited to patients with complex OC, which was defined as the presence of at least 1 of the following features: solid areas, thick papillary projections, thick septa, or purely solid echogenicity (13). Three studies investigated the intraobserver and interobserver agreements of 3DUS in distinguishing malignant from benign adnexal masses (27, 28, 37).

**Figure 2:**
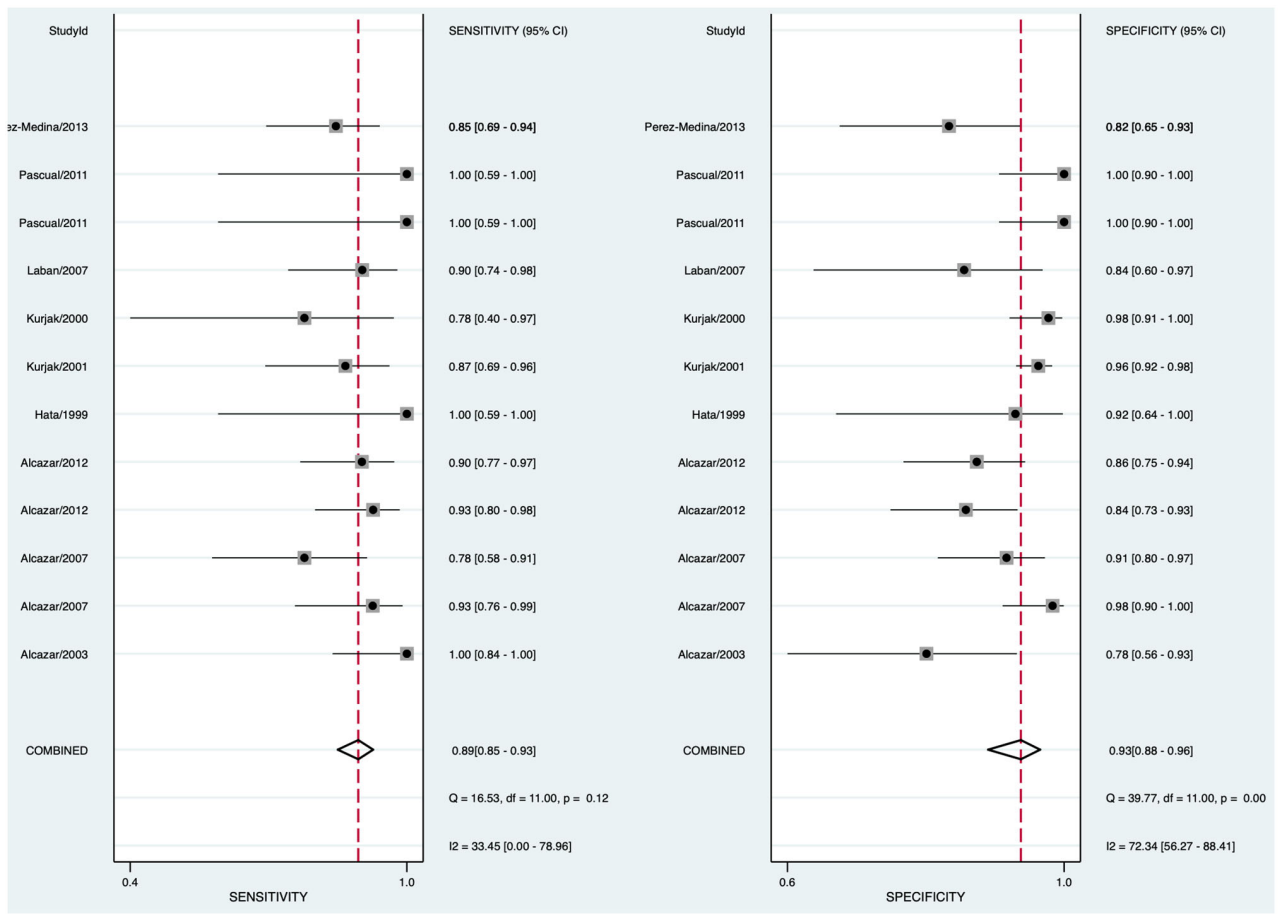
Forest plot of sensitivity and specificity for 3DUS in differentiating malignant from benign ovarian tumors.

**Figure 3:**
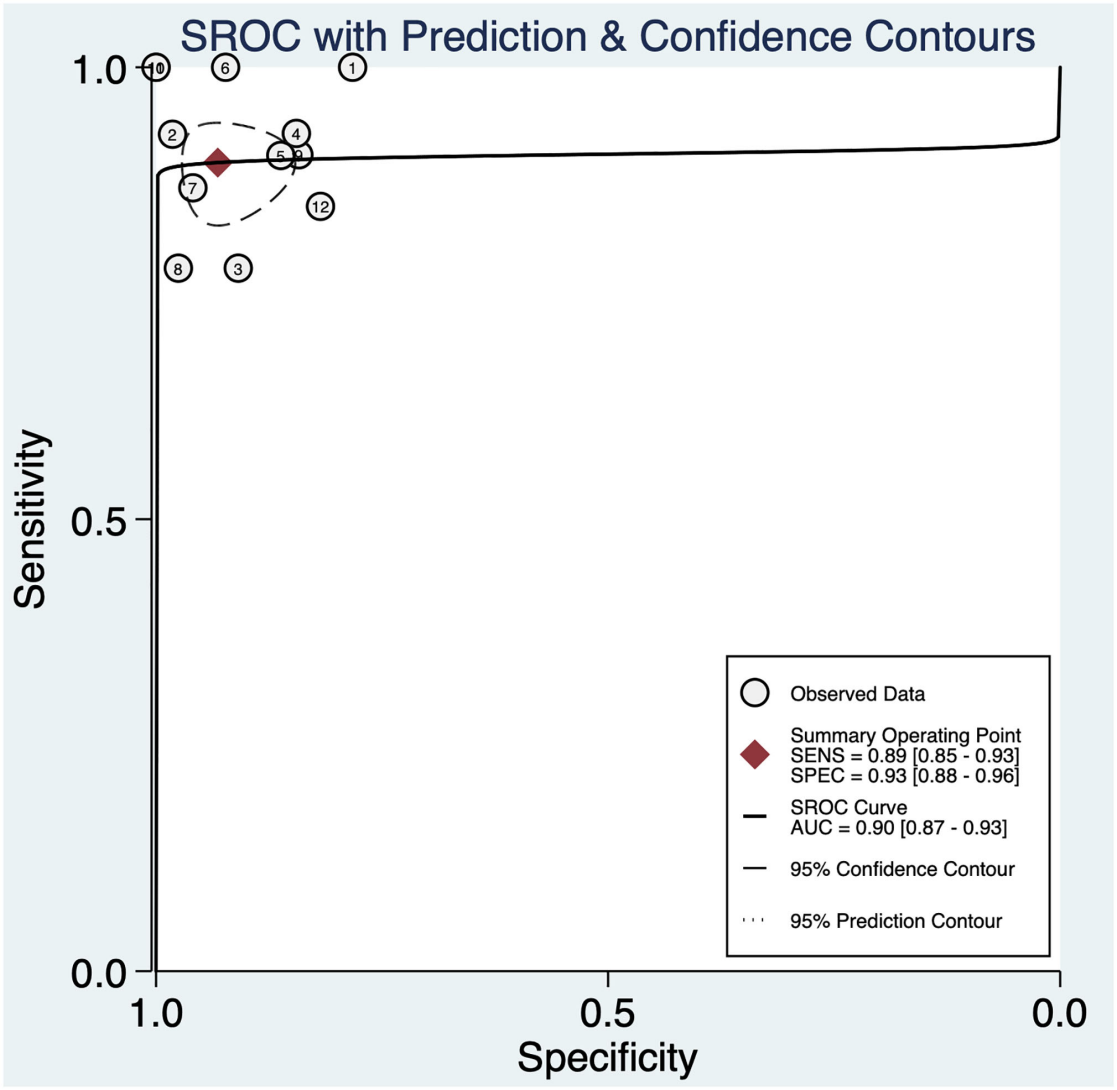
The area under the ROC curve for 3DUS in differentiating malignant from benign ovarian tumors.

The pooled sensitivity was 0.89 (95% CI: 0.85–0.93) and the pooled specificity was 0.93 (95% CI: 0.88–0.96) (**Figure 2**), indicating that 3DUS had an ability to positively and negatively differentiate OC. AUC was 0.90 (95% CI: 0.87–0.93) (**Figure 3**), suggesting that 3DUS had good accuracy in differentiating ovarian masses.

According to the forest plot, the heterogeneity of pooled sensitivity was I² = 33.45, and that of pooled specificity was I² = 72.34. There was moderate heterogeneity in pooled sensitivity and high heterogeneity in pooled specificity. To identify the source of heterogeneity, meta-regression analyses were performed. The results unveiled that the included standard of OC was the main heterogeneity for 3DUS sensitivity in OC diagnosis (*p* < 0.05) (**Supplementary Figure S1**).

A sensitivity analysis was also done to determine the impact of individual studies on the overall conclusion. The results revealed that the meta-analysis was not heavily influenced by any single study, indicating the stability of the included articles and the reliability of the findings (**Supplementary Figure S1**).

Despite some biases of the meta-analysis itself, the Deeks’ funnel plot showed no publication bias in this meta-analysis (*p* > 0.05) (**Supplementary Figure S3**).

Fagan diagram was utilized to analyze the effectiveness of 3DUS in the diagnosis of OC. Given a pre-test probability of 50%, the post-test probability of a positive result was 93% (**Supplementary Figure S4**). It implied that when the result was positive, the probability of OC was 93%, which proved that 3DUS had high clinical value in OC diagnosis. The positive likelihood ratio (PLR) was 13, and the negative likelihood ratio (NLR) was 0.11, indicating the diagnostic performance of 3DUS in OC.

### 3.2 3DPD

Eight articles on 3DPD were finally enrolled, with 671 lesions. All studies were prospective. Five articles were limited to patients with complex OC. One study investigated the intraobserver and interobserver agreements of 3DPD in distinguishing malignant from benign OC (26).

**Figure 4:**
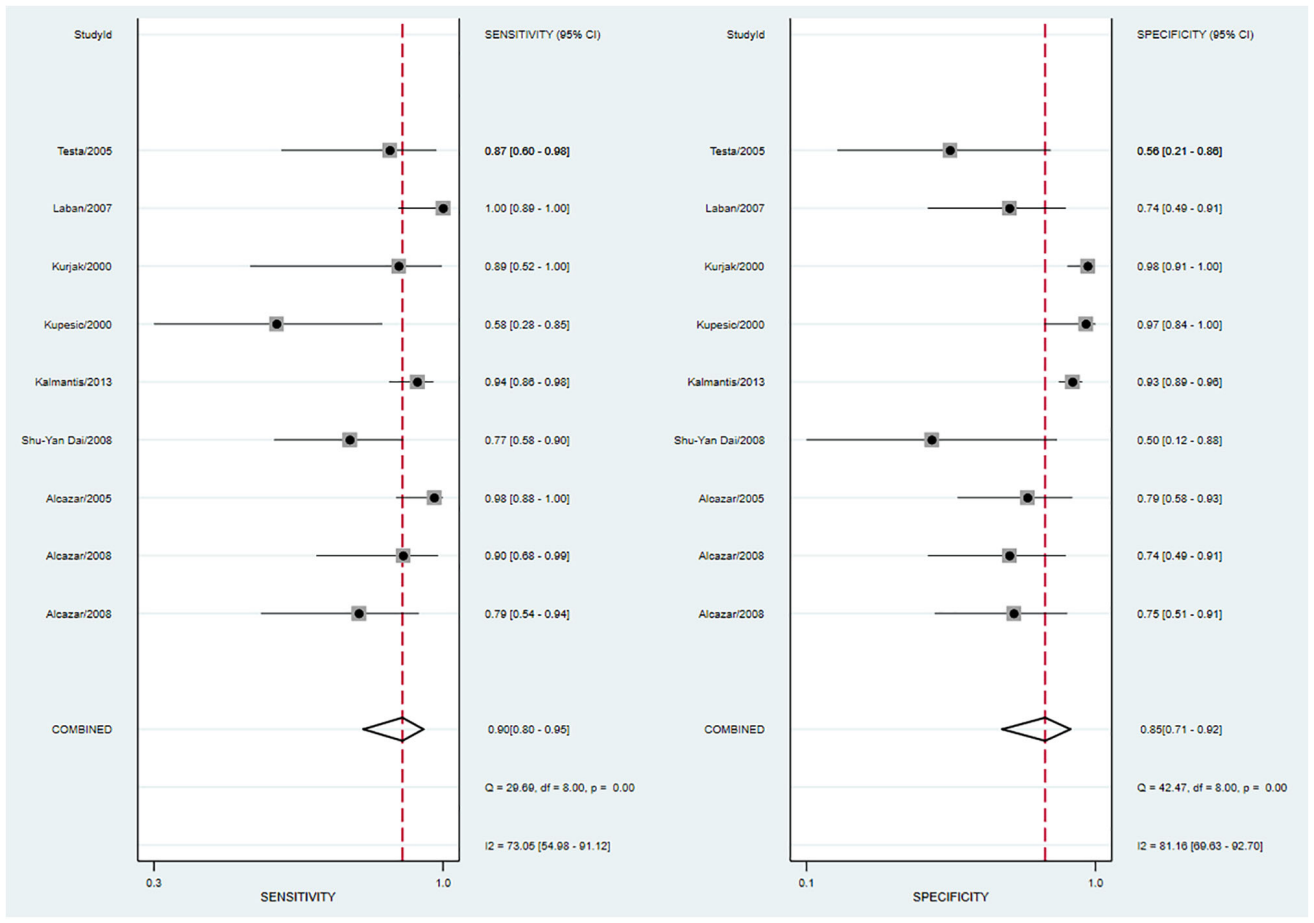
Forest plot of sensitivity and specificity for 3DPD in differentiating malignant from benign ovarian tumors.

**Figure 5:**
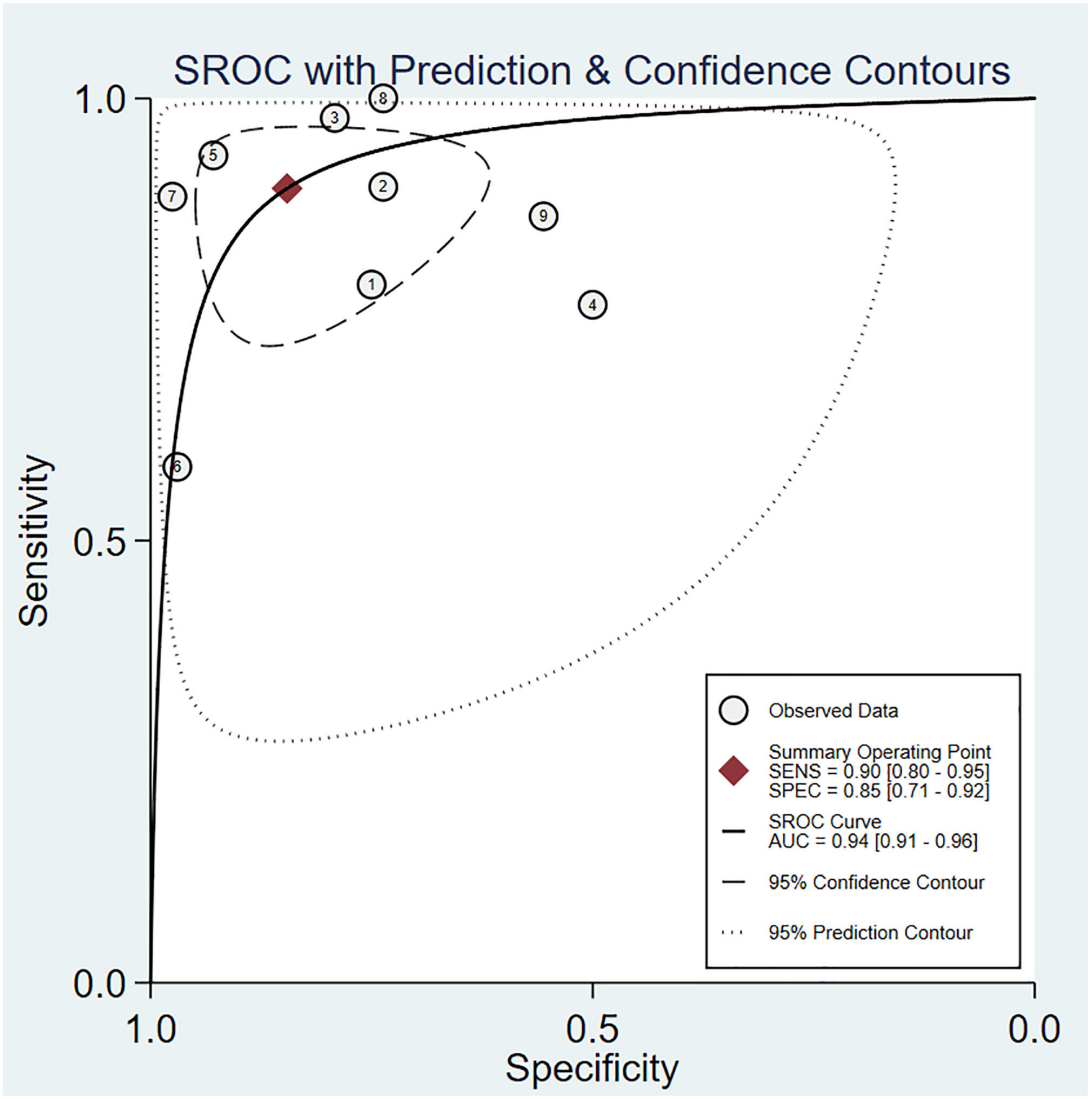
The area under the ROC curve for 3DPD in differentiating malignant from benign ovarian tumors.

The pooled sensitivity was 0.90 (95% CI: 0.80–0.95) and the pooled specificity was 0.85 (95% CI: 0.71**–**0.92) (**Figure 4**), indicating 3DPD had a respectable ability to positively and negatively differentiate OC. The AUC was 0.94 (95% CI: 0.91–0.96) (**Figure 5**), manifesting that 3DPD achieved good accuracy in differentiating OC.

According to the forest plot, the heterogeneity of pooled sensitivity was I² = 73.05, and that of pooled specificity was I² = 81.16. There was high heterogeneity in pooled sensitivity and specificity. To identify the source of heterogeneity, meta-regression analyses were performed. The results revealed that the above variables were not the sources of heterogeneity (**Supplementary Figure S5**). The main source may be the percentage of stage I and sample collection time. Sensitivity analysis revealed that no single study affected the meta-analysis results (**Supplementary Figure S6**).

The Deeks’ funnel plot suggested publication bias (*p* < 0.05), possibly because positive results were more likely to be published (**Supplementary Figure S7**).

The Fagan diagram showed that with a pre-test probability of 50%, the post-test probability of a positive result was 85% (**Supplementary Figure S8**). It indicated that when the result was positive, the probability of OC was 85%, which proved that 3DPD had good clinical value in the diagnosis of OC. The PLR was 6 and the NLR was 0.12, indicating that 3DUS cannot diagnose or exclude OC.

### 3.3 3DUS combined 3DPD

Six studies on 3DUS combined 3PDP were included, with 763 lesions. All studies were prospective. Two studies included complex OC.

**Figure 6:**
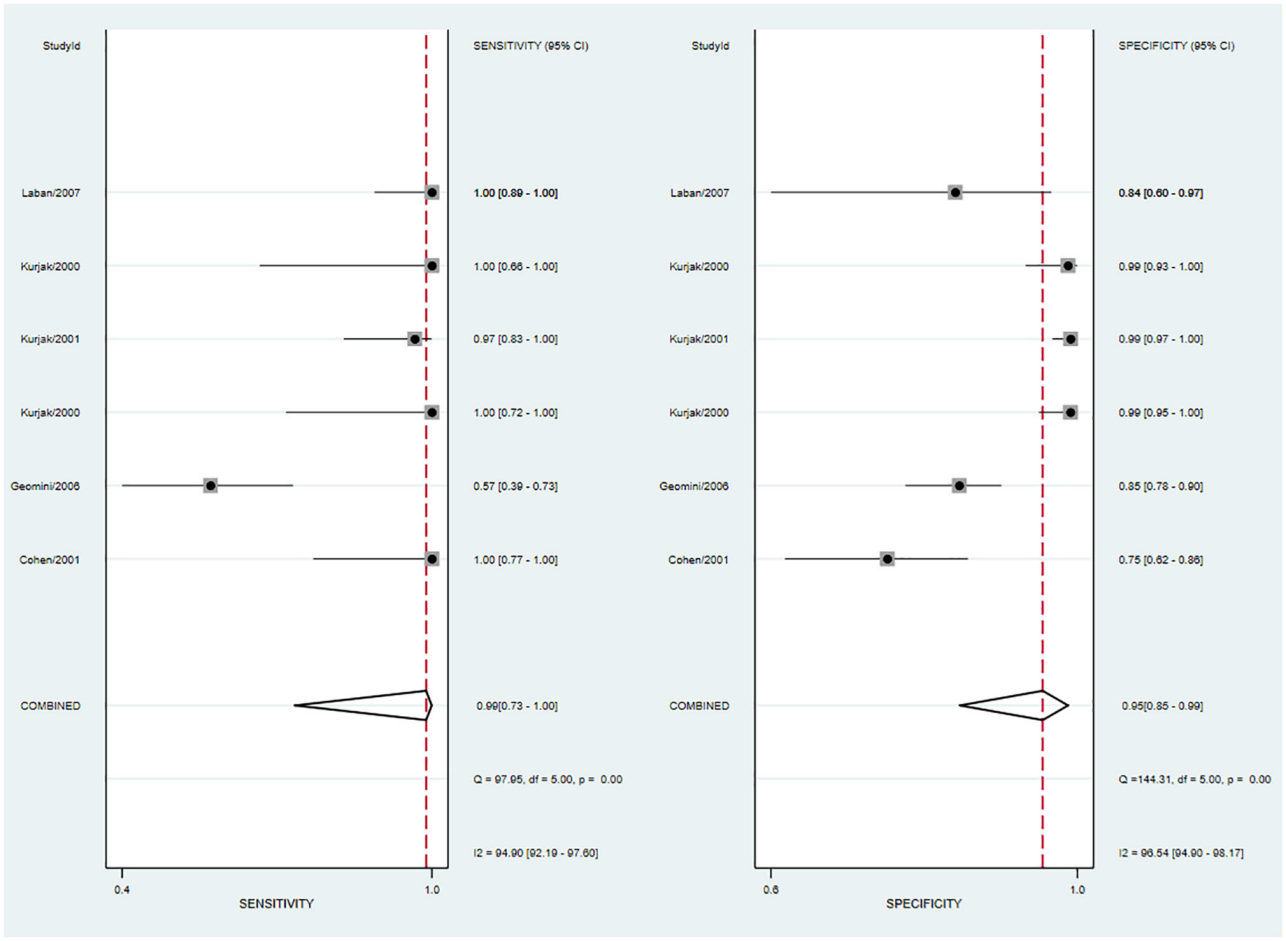
Forest plot of sensitivity and specificity for 3DUS and 3DPD in differentiating malignant from benign ovarian tumors.

**Figure 7:**
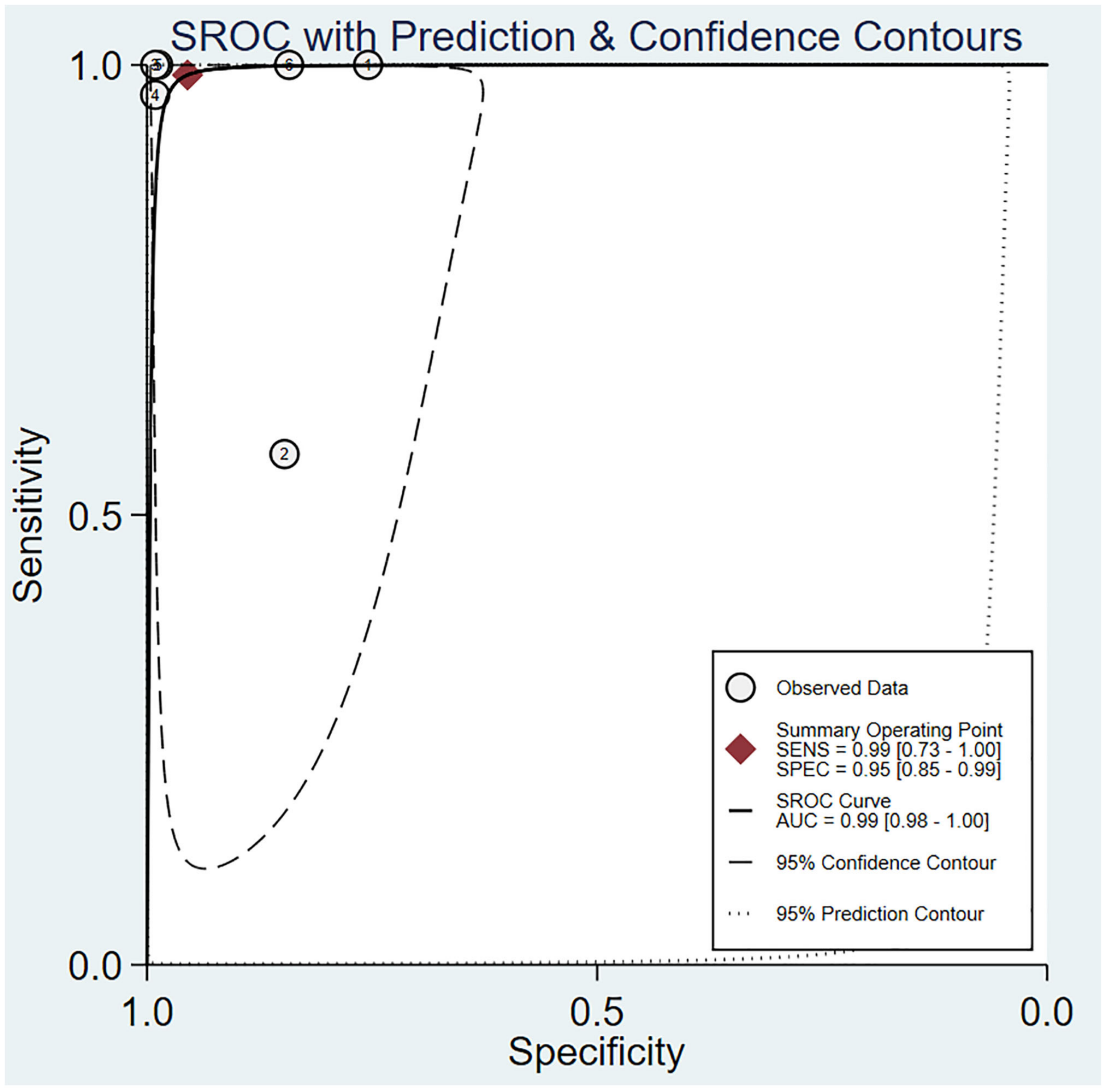
The area under the ROC curve for 3DUS and 3DPD in differentiating malignant from benign ovarian tumors.

The pooled sensitivity was 0.99 (95% CI: 0.73–1.00) and the pooled specificity was 0.95 (95% CI: 0.85–0.99; **Figure 6**), implying a significant ability to positively and negatively differentiate OC over a single diagnosis. The AUC was 0.99 (95% CI: 0.98–1.00) (**Figure 7**), indicating the excellent diagnostic accuracy of this combination tool.

The forest plot revealed that the heterogeneity of pooled sensitivity was I² = 94.40 and that of pooled specificity was I² = 96.54. There was significant heterogeneity in both pooled sensitivity and specificity. Due to the limited number of included articles, the source of heterogeneity was not analyzed.

Deeks’ funnel plot suggested no marked publication bias (*P* > 0.05) (**Supplementary Figure S9**).

The Fagan diagram showed that with a pre-test probability of 50%, the post-test probability of a positive result was 96% (**Supplementary Figure S10**). It indicated that when the result of 3DUS combined with 3DPD diagnosis was positive, the probability of OC was 96%, which proved that the combination test had very high clinical value in OC diagnosis. The PLR was 22 and the NLR was 0.01, indicating that it can diagnose and exclude OC.

## 4 Discussion

This meta-analysis assessed the diagnostic value of 3DUS, 3DPD, and 3DUS combined with 3DPD in distinguishing benign from malignant ovarian tissue. The results unveiled that 3DUS, 3DPD, and 3DUS combined with 3DPD exhibited high diagnostic values for OC. Moreover, the diagnostic value of 3DUS combined with 3DPD was superior to that of any single one.

Early detection of OC remains a significant challenge in clinical practice. To our knowledge, there is a lack of specialized diagnostic imaging techniques for OC. The typical approach for OC diagnosis is histological examination. Nevertheless, there is a growing body of research exploring the potential of 3DUS in detecting OC. This meta-analysis investigated the diagnostic performance of 3DUS in identifying OC. In this meta-analysis, 18 studies with 1,652 ovarian lesions were enrolled according to our inclusion criteria. In all studies, histologically diagnosed OC patients were considered positive. However, 41 lesions that resolved spontaneously after follow-up or showed no change in appearance by ultrasound were not confirmed through histological examination. Such a loose design may overestimate the diagnostic accuracy and lead to bias. This meta-analysis demonstrated that the AUC for diagnosing OC using 3DUS was 0.9, while the AUC using 3DPD was 0.94, and the AUC using the combined test was 0.99. All these values exceeded 0.9, indicating high accuracy. The combined test exhibited the highest diagnostic performance.

3DUS is an efficient technology for capturing digital data, enabling rapid acquisition and storage of volumetric data from ROI. Compared with traditional two-dimensional ultrasound, 3DUS offers improved visualization of the three-dimensional morphology, internal structure, and inner wall characteristics of fluid-filled areas and lesions (41). In recent years, 3DPD has attracted much attention. This technology relies on the ability to visualize the dynamics of circulating red blood cells, presenting the location of lesions and blood flow through spatially reconstructed images. Thus, it avoids the impact of vessel orientation and ultrasound detection angles, consequently minimizing the occurrence of aliasing artifacts. Additionally, 3DPD offers a broad dynamic range, enabling the detection of small vessels and low-velocity blood flow signals, thus enhancing the sensitivity to identify microvascular abnormalities (42). Furthermore, by utilizing three-dimensional imaging, 3DPD can provide a detailed and comprehensive visualization of the vascular tree or vascular network within and around lesions, achieving similar effects to vascular imaging techniques but with simple and non-invasive procedures (43). 3DUS can serve as an adjunct to routine ultrasound in cases of difficult diagnosis, and the combined application can improve the diagnostic accuracy of OC.

The presence of extensive neovascularization surrounding and within malignant ovarian tumors, marked by rapid blood flow velocities, is a common feature due to the aggressive and invasive nature of OC. In contrast, benign tumors exhibit a slow growth rate and fewer blood vessels. However, some benign and malignant tumors share overlapping areas in sonogram and blood flow classification. Some studies have noted that the blood flow intensity of OC can be influenced by local hormone levels or the luteal phase, and luteal hemorrhage can manifest as solid tumors with significant blood flow on sonograms (44). These factors all impact the accuracy of 3DUS in tumor characterization. Notably, 3DUS can neither detect surrounding and distant metastases of the tumor nor guide the clinical staging of OC. Therefore, sonographers should incorporate clinical data for comprehensive evaluation.

The heterogeneity analysis revealed different degrees of heterogeneity in 3DUS, 3DPD, and 3DUS combined with 3DPD, so the random-effects model was used for analysis. The observed heterogeneity in 3DUS may be attributed to three studies (14, 36, 38) that selected complex OC. 3DUS has its limitations. For instance, excessive gain adjustments may obscure truly solid areas, leading to the misdiagnosis of malignant lesions as benign masses. Consequently, this can contribute to higher rates of false negatives (14). Studies on 3DPD have shown differences in the proportion of stage I patients across studies, resulting in bias and heterogeneity. The sample collection time varied across studies. Five studies did not report the sample collection time, two studies (29, 32) collected data 1–2 weeks before surgery, and one study (39) collected data 2 days before surgery. In studies on 3DUS combined 3DPD for complex ovarian lesions, the specificity was lower than that of the literature not specifically mentioned this aspect. Therefore, complex ovarian lesions may be a source of heterogeneity. The number of ovarian lesions included in each literature varied greatly, ranging from 45 to 251, which may also lead to heterogeneity.

The primary advantage of our research lies in the systematic summary of available evidence on 3DUS for OC diagnosis. However, the present study has several limitations. First, the diagnostic accuracy for early stage cases (stages I–II) could not be determined due to insufficient raw data. Additionally, primary data cannot be obtained to explore changes in 3DUS values based on tumor type, histology, or stage. Second, both prospective and retrospective studies were included, thereby potentially introducing selective and recall biases. Third, the differences in the experience of ultrasound sonographers may have potentially impacted the diagnostic performance of ultrasound. Fourth, due to the limited number of included studies in the combination test, the selected factors influencing heterogeneity may be unstable and the results have limitations.

In conclusion, the current evidence suggests that 3DUS, 3DPD, and the combination of 3DUS with 3DPD have potential diagnostic value in distinguishing malignant from benign ovarian lesions. Studies incorporating both morphological and Doppler assessments have demonstrated significantly improved diagnostic accuracy compared to utilizing each modality alone. This combined approach exhibits high sensitivity and specificity. The rapid and convenient nature of 3DUS in detecting ovarian lesions suggests its potential utility for routine screening in asymptomatic high-risk populations. Further research is warranted to identify potential correlations among multiple ultrasound parameters, which may enhance the efficacy of OC detection.

## Data availability statement

The original contributions presented in the study are included in the article/**Supplementary Material**. Further inquiries can be directed to the corresponding author.

## Author contributions

YL: Conceptualization, Methodology, Writing – original draft. QZ: Formal Analysis, Investigation, Writing – original draft. FZ: Resources, Writing – review & editing. ML: Writing – review & editing. JZ: Methodology, Writing – review & editing. XC: Writing – review & editing. WY: Conceptualization, Supervision, Writing – review & editing.
