# Supplementary material for: Is three-dimensional ultrasonography a valuable diagnostic tool for patients with ovarian cancer? Systematic review and meta-analysis
Source: Front Oncol. 2024 Jul 8;14:1404426. doi: 10.3389/fonc.2024.1404426 (PMC11260635; doi:10.3389/fonc.2024.1404426)
Supplement: Supplementary file 2 [file DataSheet_2.docx]

**Supplementary material 2** search strategy

“(ovary OR ovarian OR adnexal OR famale genital OR gynecologic OR pelvic OR pelvis) AND (neoplasm OR neoplasms OR cancer OR cancers OR tumor OR tumors OR malignancy OR malignancies OR carcinoma OR carcinomas OR mass OR masses) AND (three-dimensional OR 3D) AND (ultrasonography OR ultrasound OR echotomography OR echography OR medical sonography OR ultrasonic imaging OR ultrasonographic imaging OR US OR power doppler OR PD) AND (screening OR detection OR diagnosis OR diagnoses OR examination OR evaluation)

("ovarian neoplasms"[MeSH Terms] OR ("ovarian"[All Fields] AND "neoplasms"[All Fields]) OR "ovarian neoplasms"[All Fields] OR ("ovarian"[All Fields] AND "neoplasm"[All Fields]) OR "ovarian neoplasm"[All Fields] OR ("ovarian neoplasms"[MeSH Terms] OR ("ovarian"[All Fields] AND "neoplasms"[All Fields]) OR "ovarian neoplasms"[All Fields]) OR ("ovarian neoplasms"[MeSH Terms] OR ("ovarian"[All Fields] AND "neoplasms"[All Fields]) OR "ovarian neoplasms"[All Fields] OR ("ovarian"[All Fields] AND "cancer"[All Fields]) OR "ovarian cancer"[All Fields]) OR ("ovarian neoplasms"[MeSH Terms] OR ("ovarian"[All Fields] AND "neoplasms"[All Fields]) OR "ovarian neoplasms"[All Fields] OR ("ovarian"[All Fields] AND "cancers"[All Fields]) OR "ovarian cancers"[All Fields]) OR ("ovarian neoplasms"[MeSH Terms] OR ("ovarian"[All Fields] AND "neoplasms"[All Fields]) OR "ovarian neoplasms"[All Fields] OR ("ovarian"[All Fields] AND "tumor"[All Fields]) OR "ovarian tumor"[All Fields]) OR ("ovarian neoplasms"[MeSH Terms] OR ("ovarian"[All Fields] AND "neoplasms"[All Fields]) OR "ovarian neoplasms"[All Fields] OR ("ovarian"[All Fields] AND "tumors"[All Fields]) OR "ovarian tumors"[All Fields]) OR (("ovarian"[All Fields] OR "ovarians"[All Fields]) AND ("malign"[All Fields] OR "malignance"[All Fields] OR "malignances"[All Fields] OR "malignant"[All Fields] OR "malignants"[All Fields] OR "malignities"[All Fields] OR "malignity"[All Fields] OR "malignization"[All Fields] OR "malignized"[All Fields] OR "maligns"[All Fields] OR "neoplasms"[MeSH Terms] OR "neoplasms"[All Fields] OR "malignancies"[All Fields] OR "malignancy"[All Fields])) OR (("ovarian"[All Fields] OR "ovarians"[All Fields]) AND ("malign"[All Fields] OR "malignance"[All Fields] OR "malignances"[All Fields] OR "malignant"[All Fields] OR "malignants"[All Fields] OR "malignities"[All Fields] OR "malignity"[All Fields] OR "malignization"[All Fields] OR "malignized"[All Fields] OR "maligns"[All Fields] OR "neoplasms"[MeSH Terms] OR "neoplasms"[All Fields] OR "malignancies"[All Fields] OR "malignancy"[All Fields])) OR ("ovarian neoplasms"[MeSH Terms] OR ("ovarian"[All Fields] AND "neoplasms"[All Fields]) OR "ovarian neoplasms"[All Fields] OR ("ovarian"[All Fields] AND "carcinoma"[All Fields]) OR "ovarian carcinoma"[All Fields]) OR ("ovarian neoplasms"[MeSH Terms] OR ("ovarian"[All Fields] AND "neoplasms"[All Fields]) OR "ovarian neoplasms"[All Fields] OR ("ovarian"[All Fields] AND "carcinomas"[All Fields]) OR "ovarian carcinomas"[All Fields]) OR (("ovarian"[All Fields] OR "ovarians"[All Fields]) AND ("molecular weight"[MeSH Terms] OR ("molecular"[All Fields] AND "weight"[All Fields]) OR "molecular weight"[All Fields] OR "mass"[All Fields])) OR (("ovarian"[All Fields] OR "ovarians"[All Fields]) AND ("masse"[All Fields] OR "masses"[All Fields])) OR ("ovarian neoplasms"[MeSH Terms] OR ("ovarian"[All Fields] AND "neoplasms"[All Fields]) OR "ovarian neoplasms"[All Fields] OR ("ovary"[All Fields] AND "neoplasm"[All Fields]) OR "ovary neoplasm"[All Fields] OR ("ovarian neoplasms"[MeSH Terms] OR ("ovarian"[All Fields] AND "neoplasms"[All Fields]) OR "ovarian neoplasms"[All Fields] OR ("ovary"[All Fields] AND "neoplasms"[All Fields]) OR "ovary neoplasms"[All Fields]) OR ("ovarian neoplasms"[MeSH Terms] OR ("ovarian"[All Fields] AND "neoplasms"[All Fields]) OR "ovarian neoplasms"[All Fields] OR ("ovary"[All Fields] AND "cancer"[All Fields]) OR "ovary cancer"[All Fields]) OR ("ovarian neoplasms"[MeSH Terms] OR ("ovarian"[All Fields] AND "neoplasms"[All Fields]) OR "ovarian neoplasms"[All Fields] OR ("ovary"[All Fields] AND "cancers"[All Fields]) OR "ovary cancers"[All Fields]) OR ("ovarian neoplasms"[MeSH Terms] OR ("ovarian"[All Fields] AND "neoplasms"[All Fields]) OR "ovarian neoplasms"[All Fields] OR ("ovary"[All Fields] AND "tumor"[All Fields]) OR "ovary tumor"[All Fields]) OR ("ovarian neoplasms"[MeSH Terms] OR ("ovarian"[All Fields] AND "neoplasms"[All Fields]) OR "ovarian neoplasms"[All Fields] OR ("ovary"[All Fields] AND "tumors"[All Fields]) OR "ovary tumors"[All Fields]) OR (("ovarial"[All Fields] OR "ovary"[MeSH Terms] OR "ovary"[All Fields] OR "ovaries"[All Fields] OR "ovary s"[All Fields]) AND ("malign"[All Fields] OR "malignance"[All Fields] OR "malignances"[All Fields] OR "malignant"[All Fields] OR "malignants"[All Fields] OR "malignities"[All Fields] OR "malignity"[All Fields] OR "malignization"[All Fields] OR "malignized"[All Fields] OR "maligns"[All Fields] OR "neoplasms"[MeSH Terms] OR "neoplasms"[All Fields] OR "malignancies"[All Fields] OR "malignancy"[All Fields])) OR (("ovarial"[All Fields] OR "ovary"[MeSH Terms] OR "ovary"[All Fields] OR "ovaries"[All Fields] OR "ovary s"[All Fields]) AND ("malign"[All Fields] OR "malignance"[All Fields] OR "malignances"[All Fields] OR "malignant"[All Fields] OR "malignants"[All Fields] OR "malignities"[All Fields] OR "malignity"[All Fields] OR "malignization"[All Fields] OR "malignized"[All Fields] OR "maligns"[All Fields] OR "neoplasms"[MeSH Terms] OR "neoplasms"[All Fields] OR "malignancies"[All Fields] OR "malignancy"[All Fields])) OR ("ovarian neoplasms"[MeSH Terms] OR ("ovarian"[All Fields] AND "neoplasms"[All Fields]) OR "ovarian neoplasms"[All Fields] OR ("ovary"[All Fields] AND "carcinoma"[All Fields]) OR "ovary carcinoma"[All Fields]) OR (("ovarial"[All Fields] OR "ovary"[MeSH Terms] OR "ovary"[All Fields] OR "ovaries"[All Fields] OR "ovary s"[All Fields]) AND ("carcinoma"[MeSH Terms] OR "carcinoma"[All Fields] OR "carcinomas"[All Fields] OR "carcinoma s"[All Fields])) OR (("ovarial"[All Fields] OR "ovary"[MeSH Terms] OR "ovary"[All Fields] OR "ovaries"[All Fields] OR "ovary s"[All Fields]) AND ("molecular weight"[MeSH Terms] OR ("molecular"[All Fields] AND "weight"[All Fields]) OR "molecular weight"[All Fields] OR "mass"[All Fields])) OR (("ovarial"[All Fields] OR "ovary"[MeSH Terms] OR "ovary"[All Fields] OR "ovaries"[All Fields] OR "ovary s"[All Fields]) AND ("masse"[All Fields] OR "masses"[All Fields]))) OR ((("adnex"[All Fields] OR "adnexal"[All Fields] OR "adnexes"[All Fields] OR "pelvic inflammatory disease"[MeSH Terms] OR ("pelvic"[All Fields] AND "inflammatory"[All Fields] AND "disease"[All Fields]) OR "pelvic inflammatory disease"[All Fields] OR "adnexitis"[All Fields]) AND ("neoplasm s"[All Fields] OR "neoplasms"[MeSH Terms] OR "neoplasms"[All Fields] OR "neoplasm"[All Fields])) OR (("adnex"[All Fields] OR "adnexal"[All Fields] OR "adnexes"[All Fields] OR "pelvic inflammatory disease"[MeSH Terms] OR ("pelvic"[All Fields] AND "inflammatory"[All Fields] AND "disease"[All Fields]) OR "pelvic inflammatory disease"[All Fields] OR "adnexitis"[All Fields]) AND ("neoplasm s"[All Fields] OR "neoplasms"[MeSH Terms] OR "neoplasms"[All Fields] OR "neoplasm"[All Fields])) OR (("adnex"[All Fields] OR "adnexal"[All Fields] OR "adnexes"[All Fields] OR "pelvic inflammatory disease"[MeSH Terms] OR ("pelvic"[All Fields] AND "inflammatory"[All Fields] AND "disease"[All Fields]) OR "pelvic inflammatory disease"[All Fields] OR "adnexitis"[All Fields]) AND ("cancer s"[All Fields] OR "cancerated"[All Fields] OR "canceration"[All Fields] OR "cancerization"[All Fields] OR "cancerized"[All Fields] OR "cancerous"[All Fields] OR "neoplasms"[MeSH Terms] OR "neoplasms"[All Fields] OR "cancer"[All Fields] OR "cancers"[All Fields])) OR (("adnex"[All Fields] OR "adnexal"[All Fields] OR "adnexes"[All Fields] OR "pelvic inflammatory disease"[MeSH Terms] OR ("pelvic"[All Fields] AND "inflammatory"[All Fields] AND "disease"[All Fields]) OR "pelvic inflammatory disease"[All Fields] OR "adnexitis"[All Fields]) AND ("cancer s"[All Fields] OR "cancerated"[All Fields] OR "canceration"[All Fields] OR "cancerization"[All Fields] OR "cancerized"[All Fields] OR "cancerous"[All Fields] OR "neoplasms"[MeSH Terms] OR "neoplasms"[All Fields] OR "cancer"[All Fields] OR "cancers"[All Fields])) OR (("adnex"[All Fields] OR "adnexal"[All Fields] OR "adnexes"[All Fields] OR "pelvic inflammatory disease"[MeSH Terms] OR ("pelvic"[All Fields] AND "inflammatory"[All Fields] AND "disease"[All Fields]) OR "pelvic inflammatory disease"[All Fields] OR "adnexitis"[All Fields]) AND ("cysts"[MeSH Terms] OR "cysts"[All Fields] OR "cyst"[All Fields] OR "neurofibroma"[MeSH Terms] OR "neurofibroma"[All Fields] OR "neurofibromas"[All Fields] OR "tumor s"[All Fields] OR "tumoral"[All Fields] OR "tumorous"[All Fields] OR "tumour"[All Fields] OR "neoplasms"[MeSH Terms] OR "neoplasms"[All Fields] OR "tumor"[All Fields] OR "tumour s"[All Fields] OR "tumoural"[All Fields] OR "tumourous"[All Fields] OR "tumours"[All Fields] OR "tumors"[All Fields])) OR (("adnex"[All Fields] OR "adnexal"[All Fields] OR "adnexes"[All Fields] OR "pelvic inflammatory disease"[MeSH Terms] OR ("pelvic"[All Fields] AND "inflammatory"[All Fields] AND "disease"[All Fields]) OR "pelvic inflammatory disease"[All Fields] OR "adnexitis"[All Fields]) AND ("cysts"[MeSH Terms] OR "cysts"[All Fields] OR "cyst"[All Fields] OR "neurofibroma"[MeSH Terms] OR "neurofibroma"[All Fields] OR "neurofibromas"[All Fields] OR "tumor s"[All Fields] OR "tumoral"[All Fields] OR "tumorous"[All Fields] OR "tumour"[All Fields] OR "neoplasms"[MeSH Terms] OR "neoplasms"[All Fields] OR "tumor"[All Fields] OR "tumour s"[All Fields] OR "tumoural"[All Fields] OR "tumourous"[All Fields] OR "tumours"[All Fields] OR "tumors"[All Fields])) OR (("adnex"[All Fields] OR "adnexal"[All Fields] OR "adnexes"[All Fields] OR "pelvic inflammatory disease"[MeSH Terms] OR ("pelvic"[All Fields] AND "inflammatory"[All Fields] AND "disease"[All Fields]) OR "pelvic inflammatory disease"[All Fields] OR "adnexitis"[All Fields]) AND ("malign"[All Fields] OR "malignance"[All Fields] OR "malignances"[All Fields] OR "malignant"[All Fields] OR "malignants"[All Fields] OR "malignities"[All Fields] OR "malignity"[All Fields] OR "malignization"[All Fields] OR "malignized"[All Fields] OR "maligns"[All Fields] OR "neoplasms"[MeSH Terms] OR "neoplasms"[All Fields] OR "malignancies"[All Fields] OR "malignancy"[All Fields])) OR (("adnex"[All Fields] OR "adnexal"[All Fields] OR "adnexes"[All Fields] OR "pelvic inflammatory disease"[MeSH Terms] OR ("pelvic"[All Fields] AND "inflammatory"[All Fields] AND "disease"[All Fields]) OR "pelvic inflammatory disease"[All Fields] OR "adnexitis"[All Fields]) AND ("malign"[All Fields] OR "malignance"[All Fields] OR "malignances"[All Fields] OR "malignant"[All Fields] OR "malignants"[All Fields] OR "malignities"[All Fields] OR "malignity"[All Fields] OR "malignization"[All Fields] OR "malignized"[All Fields] OR "maligns"[All Fields] OR "neoplasms"[MeSH Terms] OR "neoplasms"[All Fields] OR "malignancies"[All Fields] OR "malignancy"[All Fields])) OR ("carcinoma, skin appendage"[MeSH Terms] OR ("carcinoma"[All Fields] AND "skin"[All Fields] AND "appendage"[All Fields]) OR "skin appendage carcinoma"[All Fields] OR ("adnexal"[All Fields] AND "carcinoma"[All Fields]) OR "adnexal carcinoma"[All Fields]) OR (("adnex"[All Fields] OR "adnexal"[All Fields] OR "adnexes"[All Fields] OR "pelvic inflammatory disease"[MeSH Terms] OR ("pelvic"[All Fields] AND "inflammatory"[All Fields] AND "disease"[All Fields]) OR "pelvic inflammatory disease"[All Fields] OR "adnexitis"[All Fields]) AND ("carcinoma"[MeSH Terms] OR "carcinoma"[All Fields] OR "carcinomas"[All Fields] OR "carcinoma s"[All Fields])) OR (("adnex"[All Fields] OR "adnexal"[All Fields] OR "adnexes"[All Fields] OR "pelvic inflammatory disease"[MeSH Terms] OR ("pelvic"[All Fields] AND "inflammatory"[All Fields] AND "disease"[All Fields]) OR "pelvic inflammatory disease"[All Fields] OR "adnexitis"[All Fields]) AND ("molecular weight"[MeSH Terms] OR ("molecular"[All Fields] AND "weight"[All Fields]) OR "molecular weight"[All Fields] OR "mass"[All Fields])) OR (("adnex"[All Fields] OR "adnexal"[All Fields] OR "adnexes"[All Fields] OR "pelvic inflammatory disease"[MeSH Terms] OR ("pelvic"[All Fields] AND "inflammatory"[All Fields] AND "disease"[All Fields]) OR "pelvic inflammatory disease"[All Fields] OR "adnexitis"[All Fields]) AND ("masse"[All Fields] OR "masses"[All Fields]))) OR ("genital neoplasms, female"[MeSH Terms] OR ("genital"[All Fields] AND "neoplasms"[All Fields] AND "female"[All Fields]) OR "female genital neoplasms"[All Fields] OR ("gynecologic"[All Fields] AND "neoplasm"[All Fields]) OR "gynecologic neoplasm"[All Fields] OR ("genital neoplasms, female"[MeSH Terms] OR ("genital"[All Fields] AND "neoplasms"[All Fields] AND "female"[All Fields]) OR "female genital neoplasms"[All Fields] OR ("gynecologic"[All Fields] AND "neoplasms"[All Fields]) OR "gynecologic neoplasms"[All Fields]) OR (("gynaecologic"[All Fields] OR "gynecologic"[All Fields] OR "gynecologically"[All Fields] OR "gynecology"[MeSH Terms] OR "gynecology"[All Fields] OR "gynaecological"[All Fields] OR "gynecological"[All Fields]) AND ("cancer s"[All Fields] OR "cancerated"[All Fields] OR "canceration"[All Fields] OR "cancerization"[All Fields] OR "cancerized"[All Fields] OR "cancerous"[All Fields] OR "neoplasms"[MeSH Terms] OR "neoplasms"[All Fields] OR "cancer"[All Fields] OR "cancers"[All Fields])) OR (("gynaecologic"[All Fields] OR "gynecologic"[All Fields] OR "gynecologically"[All Fields] OR "gynecology"[MeSH Terms] OR "gynecology"[All Fields] OR "gynaecological"[All Fields] OR "gynecological"[All Fields]) AND ("cancer s"[All Fields] OR "cancerated"[All Fields] OR "canceration"[All Fields] OR "cancerization"[All Fields] OR "cancerized"[All Fields] OR "cancerous"[All Fields] OR "neoplasms"[MeSH Terms] OR "neoplasms"[All Fields] OR "cancer"[All Fields] OR "cancers"[All Fields])) OR ("genital neoplasms, female"[MeSH Terms] OR ("genital"[All Fields] AND "neoplasms"[All Fields] AND "female"[All Fields]) OR "female genital neoplasms"[All Fields] OR ("gynecologic"[All Fields] AND "tumor"[All Fields]) OR "gynecologic tumor"[All Fields]) OR (("gynaecologic"[All Fields] OR "gynecologic"[All Fields] OR "gynecologically"[All Fields] OR "gynecology"[MeSH Terms] OR "gynecology"[All Fields] OR "gynaecological"[All Fields] OR "gynecological"[All Fields]) AND ("cysts"[MeSH Terms] OR "cysts"[All Fields] OR "cyst"[All Fields] OR "neurofibroma"[MeSH Terms] OR "neurofibroma"[All Fields] OR "neurofibromas"[All Fields] OR "tumor s"[All Fields] OR "tumoral"[All Fields] OR "tumorous"[All Fields] OR "tumour"[All Fields] OR "neoplasms"[MeSH Terms] OR "neoplasms"[All Fields] OR "tumor"[All Fields] OR "tumour s"[All Fields] OR "tumoural"[All Fields] OR "tumourous"[All Fields] OR "tumours"[All Fields] OR "tumors"[All Fields])) OR (("gynaecologic"[All Fields] OR "gynecologic"[All Fields] OR "gynecologically"[All Fields] OR "gynecology"[MeSH Terms] OR "gynecology"[All Fields] OR "gynaecological"[All Fields] OR "gynecological"[All Fields]) AND ("malign"[All Fields] OR "malignance"[All Fields] OR "malignances"[All Fields] OR "malignant"[All Fields] OR "malignants"[All Fields] OR "malignities"[All Fields] OR "malignity"[All Fields] OR "malignization"[All Fields] OR "malignized"[All Fields] OR "maligns"[All Fields] OR "neoplasms"[MeSH Terms] OR "neoplasms"[All Fields] OR "malignancies"[All Fields] OR "malignancy"[All Fields])) OR (("gynaecologic"[All Fields] OR "gynecologic"[All Fields] OR "gynecologically"[All Fields] OR "gynecology"[MeSH Terms] OR "gynecology"[All Fields] OR "gynaecological"[All Fields] OR "gynecological"[All Fields]) AND ("malign"[All Fields] OR "malignance"[All Fields] OR "malignances"[All Fields] OR "malignant"[All Fields] OR "malignants"[All Fields] OR "malignities"[All Fields] OR "malignity"[All Fields] OR "malignization"[All Fields] OR "malignized"[All Fields] OR "maligns"[All Fields] OR "neoplasms"[MeSH Terms] OR "neoplasms"[All Fields] OR "malignancies"[All Fields] OR "malignancy"[All Fields])) OR (("gynaecologic"[All Fields] OR "gynecologic"[All Fields] OR "gynecologically"[All Fields] OR "gynecology"[MeSH Terms] OR "gynecology"[All Fields] OR "gynaecological"[All Fields] OR "gynecological"[All Fields]) AND ("carcinoma"[MeSH Terms] OR "carcinoma"[All Fields] OR "carcinomas"[All Fields] OR "carcinoma s"[All Fields])) OR (("gynaecologic"[All Fields] OR "gynecologic"[All Fields] OR "gynecologically"[All Fields] OR "gynecology"[MeSH Terms] OR "gynecology"[All Fields] OR "gynaecological"[All Fields] OR "gynecological"[All Fields]) AND ("carcinoma"[MeSH Terms] OR "carcinoma"[All Fields] OR "carcinomas"[All Fields] OR "carcinoma s"[All Fields])) OR (("gynaecologic"[All Fields] OR "gynecologic"[All Fields] OR "gynecologically"[All Fields] OR "gynecology"[MeSH Terms] OR "gynecology"[All Fields] OR "gynaecological"[All Fields] OR "gynecological"[All Fields]) AND ("molecular weight"[MeSH Terms] OR ("molecular"[All Fields] AND "weight"[All Fields]) OR "molecular weight"[All Fields] OR "mass"[All Fields])) OR (("gynaecologic"[All Fields] OR "gynecologic"[All Fields] OR "gynecologically"[All Fields] OR "gynecology"[MeSH Terms] OR "gynecology"[All Fields] OR "gynaecological"[All Fields] OR "gynecological"[All Fields]) AND ("masse"[All Fields] OR "masses"[All Fields])) OR ("genital neoplasms, female"[MeSH Terms] OR ("genital"[All Fields] AND "neoplasms"[All Fields] AND "female"[All Fields]) OR "female genital neoplasms"[All Fields] OR ("female"[All Fields] AND "genital"[All Fields] AND "neoplasm"[All Fields]) OR "female genital neoplasm"[All Fields] OR ("genital neoplasms, female"[MeSH Terms] OR ("genital"[All Fields] AND "neoplasms"[All Fields] AND "female"[All Fields]) OR "female genital neoplasms"[All Fields] OR ("female"[All Fields] AND "genital"[All Fields] AND "neoplasms"[All Fields])) OR (("genitalia, female"[MeSH Terms] OR ("genitalia"[All Fields] AND "female"[All Fields]) OR "female genitalia"[All Fields] OR ("female"[All Fields] AND "genital"[All Fields]) OR "female genital"[All Fields]) AND ("cancer s"[All Fields] OR "cancerated"[All Fields] OR "canceration"[All Fields] OR "cancerization"[All Fields] OR "cancerized"[All Fields] OR "cancerous"[All Fields] OR "neoplasms"[MeSH Terms] OR "neoplasms"[All Fields] OR "cancer"[All Fields] OR "cancers"[All Fields])) OR (("genitalia, female"[MeSH Terms] OR ("genitalia"[All Fields] AND "female"[All Fields]) OR "female genitalia"[All Fields] OR ("female"[All Fields] AND "genital"[All Fields]) OR "female genital"[All Fields]) AND ("cancer s"[All Fields] OR "cancerated"[All Fields] OR "canceration"[All Fields] OR "cancerization"[All Fields] OR "cancerized"[All Fields] OR "cancerous"[All Fields] OR "neoplasms"[MeSH Terms] OR "neoplasms"[All Fields] OR "cancer"[All Fields] OR "cancers"[All Fields])) OR (("genitalia, female"[MeSH Terms] OR ("genitalia"[All Fields] AND "female"[All Fields]) OR "female genitalia"[All Fields] OR ("female"[All Fields] AND "genital"[All Fields]) OR "female genital"[All Fields]) AND ("cysts"[MeSH Terms] OR "cysts"[All Fields] OR "cyst"[All Fields] OR "neurofibroma"[MeSH Terms] OR "neurofibroma"[All Fields] OR "neurofibromas"[All Fields] OR "tumor s"[All Fields] OR "tumoral"[All Fields] OR "tumorous"[All Fields] OR "tumour"[All Fields] OR "neoplasms"[MeSH Terms] OR "neoplasms"[All Fields] OR "tumor"[All Fields] OR "tumour s"[All Fields] OR "tumoural"[All Fields] OR "tumourous"[All Fields] OR "tumours"[All Fields] OR "tumors"[All Fields])) OR (("genitalia, female"[MeSH Terms] OR ("genitalia"[All Fields] AND "female"[All Fields]) OR "female genitalia"[All Fields] OR ("female"[All Fields] AND "genital"[All Fields]) OR "female genital"[All Fields]) AND ("cysts"[MeSH Terms] OR "cysts"[All Fields] OR "cyst"[All Fields] OR "neurofibroma"[MeSH Terms] OR "neurofibroma"[All Fields] OR "neurofibromas"[All Fields] OR "tumor s"[All Fields] OR "tumoral"[All Fields] OR "tumorous"[All Fields] OR "tumour"[All Fields] OR "neoplasms"[MeSH Terms] OR "neoplasms"[All Fields] OR "tumor"[All Fields] OR "tumour s"[All Fields] OR "tumoural"[All Fields] OR "tumourous"[All Fields] OR "tumours"[All Fields] OR "tumors"[All Fields])) OR (("genitalia, female"[MeSH Terms] OR ("genitalia"[All Fields] AND "female"[All Fields]) OR "female genitalia"[All Fields] OR ("female"[All Fields] AND "genital"[All Fields]) OR "female genital"[All Fields]) AND ("malign"[All Fields] OR "malignance"[All Fields] OR "malignances"[All Fields] OR "malignant"[All Fields] OR "malignants"[All Fields] OR "malignities"[All Fields] OR "malignity"[All Fields] OR "malignization"[All Fields] OR "malignized"[All Fields] OR "maligns"[All Fields] OR "neoplasms"[MeSH Terms] OR "neoplasms"[All Fields] OR "malignancies"[All Fields] OR "malignancy"[All Fields])) OR (("genitalia, female"[MeSH Terms] OR ("genitalia"[All Fields] AND "female"[All Fields]) OR "female genitalia"[All Fields] OR ("female"[All Fields] AND "genital"[All Fields]) OR "female genital"[All Fields]) AND ("malign"[All Fields] OR "malignance"[All Fields] OR "malignances"[All Fields] OR "malignant"[All Fields] OR "malignants"[All Fields] OR "malignities"[All Fields] OR "malignity"[All Fields] OR "malignization"[All Fields] OR "malignized"[All Fields] OR "maligns"[All Fields] OR "neoplasms"[MeSH Terms] OR "neoplasms"[All Fields] OR "malignancies"[All Fields] OR "malignancy"[All Fields])) OR (("genitalia, female"[MeSH Terms] OR ("genitalia"[All Fields] AND "female"[All Fields]) OR "female genitalia"[All Fields] OR ("female"[All Fields] AND "genital"[All Fields]) OR "female genital"[All Fields]) AND ("carcinoma"[MeSH Terms] OR "carcinoma"[All Fields] OR "carcinomas"[All Fields] OR "carcinoma s"[All Fields])) OR (("genitalia, female"[MeSH Terms] OR ("genitalia"[All Fields] AND "female"[All Fields]) OR "female genitalia"[All Fields] OR ("female"[All Fields] AND "genital"[All Fields]) OR "female genital"[All Fields]) AND ("carcinoma"[MeSH Terms] OR "carcinoma"[All Fields] OR "carcinomas"[All Fields] OR "carcinoma s"[All Fields])) OR (("genitalia, female"[MeSH Terms] OR ("genitalia"[All Fields] AND "female"[All Fields]) OR "female genitalia"[All Fields] OR ("female"[All Fields] AND "genital"[All Fields]) OR "female genital"[All Fields]) AND ("molecular weight"[MeSH Terms] OR ("molecular"[All Fields] AND "weight"[All Fields]) OR "molecular weight"[All Fields] OR "mass"[All Fields])) OR (("genitalia, female"[MeSH Terms] OR ("genitalia"[All Fields] AND "female"[All Fields]) OR "female genitalia"[All Fields] OR ("female"[All Fields] AND "genital"[All Fields]) OR "female genital"[All Fields]) AND ("masse"[All Fields] OR "masses"[All Fields])))) OR ("pelvic neoplasms"[MeSH Terms] OR ("pelvic"[All Fields] AND "neoplasms"[All Fields]) OR "pelvic neoplasms"[All Fields] OR ("pelvic"[All Fields] AND "neoplasm"[All Fields]) OR "pelvic neoplasm"[All Fields] OR ("pelvic neoplasms"[MeSH Terms] OR ("pelvic"[All Fields] AND "neoplasms"[All Fields]) OR "pelvic neoplasms"[All Fields]) OR ("pelvic neoplasms"[MeSH Terms] OR ("pelvic"[All Fields] AND "neoplasms"[All Fields]) OR "pelvic neoplasms"[All Fields] OR ("pelvic"[All Fields] AND "cancer"[All Fields]) OR "pelvic cancer"[All Fields]) OR ("pelvic neoplasms"[MeSH Terms] OR ("pelvic"[All Fields] AND "neoplasms"[All Fields]) OR "pelvic neoplasms"[All Fields] OR ("pelvic"[All Fields] AND "cancers"[All Fields]) OR "pelvic cancers"[All Fields]) OR ("pelvic neoplasms"[MeSH Terms] OR ("pelvic"[All Fields] AND "neoplasms"[All Fields]) OR "pelvic neoplasms"[All Fields] OR ("pelvic"[All Fields] AND "tumor"[All Fields]) OR "pelvic tumor"[All Fields]) OR ("pelvic neoplasms"[MeSH Terms] OR ("pelvic"[All Fields] AND "neoplasms"[All Fields]) OR "pelvic neoplasms"[All Fields] OR ("pelvic"[All Fields] AND "tumors"[All Fields]) OR "pelvic tumors"[All Fields]) OR (("pelvics"[All Fields] OR "pelvis"[MeSH Terms] OR "pelvis"[All Fields] OR "pelvic"[All Fields]) AND ("malign"[All Fields] OR "malignance"[All Fields] OR "malignances"[All Fields] OR "malignant"[All Fields] OR "malignants"[All Fields] OR "malignities"[All Fields] OR "malignity"[All Fields] OR "malignization"[All Fields] OR "malignized"[All Fields] OR "maligns"[All Fields] OR "neoplasms"[MeSH Terms] OR "neoplasms"[All Fields] OR "malignancies"[All Fields] OR "malignancy"[All Fields])) OR (("pelvics"[All Fields] OR "pelvis"[MeSH Terms] OR "pelvis"[All Fields] OR "pelvic"[All Fields]) AND ("malign"[All Fields] OR "malignance"[All Fields] OR "malignances"[All Fields] OR "malignant"[All Fields] OR "malignants"[All Fields] OR "malignities"[All Fields] OR "malignity"[All Fields] OR "malignization"[All Fields] OR "malignized"[All Fields] OR "maligns"[All Fields] OR "neoplasms"[MeSH Terms] OR "neoplasms"[All Fields] OR "malignancies"[All Fields] OR "malignancy"[All Fields])) OR (("pelvics"[All Fields] OR "pelvis"[MeSH Terms] OR "pelvis"[All Fields] OR "pelvic"[All Fields]) AND ("carcinoma"[MeSH Terms] OR "carcinoma"[All Fields] OR "carcinomas"[All Fields] OR "carcinoma s"[All Fields])) OR (("pelvics"[All Fields] OR "pelvis"[MeSH Terms] OR "pelvis"[All Fields] OR "pelvic"[All Fields]) AND ("carcinoma"[MeSH Terms] OR "carcinoma"[All Fields] OR "carcinomas"[All Fields] OR "carcinoma s"[All Fields])) OR (("pelvics"[All Fields] OR "pelvis"[MeSH Terms] OR "pelvis"[All Fields] OR "pelvic"[All Fields]) AND ("molecular weight"[MeSH Terms] OR ("molecular"[All Fields] AND "weight"[All Fields]) OR "molecular weight"[All Fields] OR "mass"[All Fields])) OR (("pelvics"[All Fields] OR "pelvis"[MeSH Terms] OR "pelvis"[All Fields] OR "pelvic"[All Fields]) AND ("masse"[All Fields] OR "masses"[All Fields])) OR ("pelvic neoplasms"[MeSH Terms] OR ("pelvic"[All Fields] AND "neoplasms"[All Fields]) OR "pelvic neoplasms"[All Fields] OR ("pelvis"[All Fields] AND "neoplasm"[All Fields]) OR "pelvis neoplasm"[All Fields] OR ("pelvic neoplasms"[MeSH Terms] OR ("pelvic"[All Fields] AND "neoplasms"[All Fields]) OR "pelvic neoplasms"[All Fields] OR ("pelvis"[All Fields] AND "neoplasms"[All Fields]) OR "pelvis neoplasms"[All Fields]) OR ("pelvic neoplasms"[MeSH Terms] OR ("pelvic"[All Fields] AND "neoplasms"[All Fields]) OR "pelvic neoplasms"[All Fields] OR ("pelvis"[All Fields] AND "cancer"[All Fields]) OR "pelvis cancer"[All Fields]) OR ("pelvic neoplasms"[MeSH Terms] OR ("pelvic"[All Fields] AND "neoplasms"[All Fields]) OR "pelvic neoplasms"[All Fields] OR ("pelvis"[All Fields] AND "cancers"[All Fields]) OR "pelvis cancers"[All Fields]) OR ("pelvic neoplasms"[MeSH Terms] OR ("pelvic"[All Fields] AND "neoplasms"[All Fields]) OR "pelvic neoplasms"[All Fields] OR ("pelvis"[All Fields] AND "tumor"[All Fields]) OR "pelvis tumor"[All Fields]) OR ("pelvic neoplasms"[MeSH Terms] OR ("pelvic"[All Fields] AND "neoplasms"[All Fields]) OR "pelvic neoplasms"[All Fields] OR ("pelvis"[All Fields] AND "tumors"[All Fields]) OR "pelvis tumors"[All Fields]) OR (("pelvi"[All Fields] OR "pelvis"[MeSH Terms] OR "pelvis"[All Fields]) AND ("malign"[All Fields] OR "malignance"[All Fields] OR "malignances"[All Fields] OR "malignant"[All Fields] OR "malignants"[All Fields] OR "malignities"[All Fields] OR "malignity"[All Fields] OR "malignization"[All Fields] OR "malignized"[All Fields] OR "maligns"[All Fields] OR "neoplasms"[MeSH Terms] OR "neoplasms"[All Fields] OR "malignancies"[All Fields] OR "malignancy"[All Fields])) OR (("pelvi"[All Fields] OR "pelvis"[MeSH Terms] OR "pelvis"[All Fields]) AND ("malign"[All Fields] OR "malignance"[All Fields] OR "malignances"[All Fields] OR "malignant"[All Fields] OR "malignants"[All Fields] OR "malignities"[All Fields] OR "malignity"[All Fields] OR "malignization"[All Fields] OR "malignized"[All Fields] OR "maligns"[All Fields] OR "neoplasms"[MeSH Terms] OR "neoplasms"[All Fields] OR "malignancies"[All Fields] OR "malignancy"[All Fields])) OR (("pelvi"[All Fields] OR "pelvis"[MeSH Terms] OR "pelvis"[All Fields]) AND ("carcinoma"[MeSH Terms] OR "carcinoma"[All Fields] OR "carcinomas"[All Fields] OR "carcinoma s"[All Fields])) OR (("pelvi"[All Fields] OR "pelvis"[MeSH Terms] OR "pelvis"[All Fields]) AND ("carcinoma"[MeSH Terms] OR "carcinoma"[All Fields] OR "carcinomas"[All Fields] OR "carcinoma s"[All Fields])) OR (("pelvi"[All Fields] OR "pelvis"[MeSH Terms] OR "pelvis"[All Fields]) AND ("molecular weight"[MeSH Terms] OR ("molecular"[All Fields] AND "weight"[All Fields]) OR "molecular weight"[All Fields] OR "mass"[All Fields])) OR (("pelvi"[All Fields] OR "pelvis"[MeSH Terms] OR "pelvis"[All Fields]) AND ("masse"[All Fields] OR "masses"[All Fields]))))) AND ((("three-dimensional"[All Fields] OR "3D"[All Fields]) AND ("diagnostic imaging"[MeSH Subheading] OR ("diagnostic"[All Fields] AND "imaging"[All Fields]) OR "diagnostic imaging"[All Fields] OR "ultrasonography"[All Fields] OR "ultrasonography"[MeSH Terms] OR "ultrasonographies"[All Fields] OR ("diagnostic imaging"[MeSH Subheading] OR ("diagnostic"[All Fields] AND "imaging"[All Fields]) OR "diagnostic imaging"[All Fields] OR "ultrasound"[All Fields] OR "ultrasonography"[MeSH Terms] OR "ultrasonography"[All Fields] OR "ultrasonics"[MeSH Terms] OR "ultrasonics"[All Fields] OR "ultrasounds"[All Fields] OR "ultrasound s"[All Fields]) OR ("diagnostic imaging"[MeSH Subheading] OR ("diagnostic"[All Fields] AND "imaging"[All Fields]) OR "diagnostic imaging"[All Fields] OR "echotomography"[All Fields] OR "ultrasonography"[MeSH Terms] OR "ultrasonography"[All Fields]) OR ("diagnostic imaging"[MeSH Subheading] OR ("diagnostic"[All Fields] AND "imaging"[All Fields]) OR "diagnostic imaging"[All Fields] OR "echography"[All Fields] OR "ultrasonography"[MeSH Terms] OR "ultrasonography"[All Fields] OR "echographies"[All Fields]) OR ("ultrasonography"[MeSH Terms] OR "ultrasonography"[All Fields] OR ("medical"[All Fields] AND "sonography"[All Fields]) OR "medical sonography"[All Fields]) OR ("ultrasonography"[MeSH Terms] OR "ultrasonography"[All Fields] OR ("ultrasonic"[All Fields] AND "imaging"[All Fields]) OR "ultrasonic imaging"[All Fields]) OR ("ultrasonography"[MeSH Terms] OR "ultrasonography"[All Fields] OR ("ultrasonographic"[All Fields] AND "imaging"[All Fields]) OR "ultrasonographic imaging"[All Fields]) OR "US"[All Fields])) OR (("three-dimensional"[All Fields] OR "3D"[All Fields]) AND ((("power, psychological"[MeSH Terms] OR ("power"[All Fields] AND "psychological"[All Fields]) OR "psychological power"[All Fields] OR "power"[All Fields] OR "powered"[All Fields] OR "powers"[All Fields] OR "powering"[All Fields]) AND ("doppler"[All Fields] OR "doppler s"[All Fields] OR "dopplers"[All Fields])) OR ("pharmacology"[MeSH Subheading] OR "pharmacology"[All Fields] OR "pd"[All Fields])))) AND ("diagnosable"[All Fields] OR "diagnosi"[All Fields] OR "diagnosis"[MeSH Terms] OR "diagnosis"[All Fields] OR "diagnose"[All Fields] OR "diagnosed"[All Fields] OR "diagnoses"[All Fields] OR "diagnosing"[All Fields] OR "diagnosis"[MeSH Subheading] OR ("diagnosable"[All Fields] OR "diagnosi"[All Fields] OR "diagnosis"[MeSH Terms] OR "diagnosis"[All Fields] OR "diagnose"[All Fields] OR "diagnosed"[All Fields] OR "diagnoses"[All Fields] OR "diagnosing"[All Fields] OR "diagnosis"[MeSH Subheading]) OR ("diagnosis"[MeSH Subheading] OR "diagnosis"[All Fields] OR "screening"[All Fields] OR "mass screening"[MeSH Terms] OR ("mass"[All Fields] AND "screening"[All Fields]) OR "mass screening"[All Fields] OR "early detection of cancer"[MeSH Terms] OR ("early"[All Fields] AND "detection"[All Fields] AND "cancer"[All Fields]) OR "early detection of cancer"[All Fields] OR "screen"[All Fields] OR "screenings"[All Fields] OR "screened"[All Fields] OR "screens"[All Fields]) OR ("detect"[All Fields] OR "detectabilities"[All Fields] OR "detectability"[All Fields] OR "detectable"[All Fields] OR "detectables"[All Fields] OR "detectably"[All Fields] OR "detected"[All Fields] OR "detectible"[All Fields] OR "detecting"[All Fields] OR "detection"[All Fields] OR "detections"[All Fields] OR "detects"[All Fields]) OR ("examination s"[All Fields] OR "examinator"[All Fields] OR "examinators"[All Fields] OR "examiner"[All Fields] OR "examiner s"[All Fields] OR "examiners"[All Fields] OR "physical examination"[MeSH Terms] OR ("physical"[All Fields] AND "examination"[All Fields]) OR "physical examination"[All Fields] OR "examination"[All Fields] OR "examinations"[All Fields]) OR ("evaluability"[All Fields] OR "evaluate"[All Fields] OR "evaluated"[All Fields] OR "evaluates"[All Fields] OR "evaluating"[All Fields] OR "evaluation"[All Fields] OR "evaluation s"[All Fields] OR "evaluations"[All Fields] OR "evaluative"[All Fields] OR "evaluatively"[All Fields] OR "evaluatives"[All Fields] OR "evaluator"[All Fields] OR "evaluator s"[All Fields] OR "evaluators"[All Fields]))
